# Supplementary material for: MetaRibo-Seq measures translation in microbiomes
Source: Nat Commun. 2020 Jun 29;11:3268. doi: 10.1038/s41467-020-17081-z (PMC7324362; doi:10.1038/s41467-020-17081-z)
Supplement: Supplementary file 10 — Supplementary Data 7 [file 41467_2020_17081_MOESM10_ESM.zip › File2/Confidence_High_Taxonomy/60927_out.krona.html]

Javascript must be enabled to view this page.

members
magnitude
magnitudeUnassigned
count
unassigned
taxon
rank

60927\_out

5

2
superkingdom
5

1239
phylum
5

class
3
186801

order
3
186802

3
family
186803

265975
genus
3

species
2
1501332

SRS016815\_contig\_number\_5422SRS075765\_contig\_number\_6089


SRS098498\_contig\_number\_contig-100\_3267.270375
species
1
1501329

526524
class
2

2
order
526525

family
2
128827


SRS015038\_contig\_number\_contig-100\_260.260SRS077633\_contig\_number\_contig-100\_3432.152765
331630
2
species
